# Visual acuity and anatomical changes following vitrectomy for epiretinal membrane foveoschisis: a case series

**DOI:** 10.1186/s12886-021-02203-y

**Published:** 2021-12-15

**Authors:** Chaiyaphot Photcharapongsakul, Susama Chokesuwattanaskul, Janejit Choovuthayakorn, Voraporn Chaikitmongkol, Paradee Kunavisarut, Nawat Watanachai, Direk Patikulsila

**Affiliations:** Department of Ophthalmology, Faculty of Medicine, Chiang Mai University, 110 Intavaroros Road, Maung, Chiang Mai, 50200 Thailand

**Keywords:** ERM Foveoschisis, Visual outcomes, Anatomical outcomes

## Abstract

**Purpose:**

To evaluate the visual outcome and macular anatomic structures on spectral-domain optical coherence tomography (SD-OCT) of patients with epiretinal membrane (ERM) foveoschisis who underwent vitrectomy.

**Methods:**

A retrospective cohort, interventional, case series.

**Participants:**

Fourteen patients (14 eyes) with central vision loss from an ERM foveoschisis underwent vitrectomy at Chiang Mai University Hospital from 2017 to 2018 and had a follow-up period of 12 months.

**Interventions:**

The 23G vitrectomy with ERM and internal limiting membrane (ILM) peeling was performed by a single surgeon.

**Main outcomes:**

Best-corrected visual acuity (BCVA) and anatomic appearance on SD-OCT were assessed at the time of preoperative evaluations and post-operative follow-ups at 1, 3, 6, and 12 months.

**Results:**

Fourteen patients with a mean (SD) age of 67.9 (7.9) years and a mean (SD) visual acuity (VA) of 0.6 (0.1) LogMAR units were included in this study. Significant VA improvements were observed at the 3-month (0.43 (0.14) LogMAR unit), 6-month (0.45 (0.16) LogMAR unit) and 12-month (0.37 (0.21) LogMAR unit) post-operative visits compared to baseline, all with *P*-values < 0.001. At month 12, there were vision improvements of ≥3 lines in 8 (57.2%) patients, vision improvements of 1 or 2 lines in 2 (14.3%) patients, vision remained at the same line of pre-operation in 3 (21.4%) patients, and vision decreased by 1 line in 1 (7.1%) patient. Regarding the anatomical outcomes, 13 (92.9%) patients achieved anatomical foveal restoration, while one had persistent intraretinal schisis at the 12-month follow-up. The median time to achieve a foveal restoration was 3 months. No significant visual impairments were observed post-operatively.

**Conclusion:**

In patients with central vision loss from ERM foveoschisis, vitrectomy with ILM stripping tended to improve both visual and anatomical outcomes.

## Introduction

In the past, Gass and Allen described lamellar macular hole (LMH) as a round reddish partial-thickness macular lesion observed by slit-lamp biomicroscopic examination in patients with pseudophakic cystoid macular edema [1]. Later on, with advanced retinal imaging technologies, including spectral-domain optical coherence tomography (SD-OCT), more detailed retinal microstructural features could be visualized. Consequently, several distinct irregular foveal contours from OCT have been categorized regarding their possible underlying pathophysiologies [2–6].

Controversies have been reported regarding the natural history, surgical outcomes and prognostic predictors of these irregular foveal contours due to inconsistent definition criteria [7–9]. To facilitate universal understanding between studies, a panel of international retinal experts has recently proposed a consensus OCT-based definition for irregular inner foveal contour lesions. LMH is characterised as an irregular inner foveal contour with disruption of the inner retinal layer, having an undermined foveal cavitation edge, and presumed to be related with a degenerative mechanism. However, a condition associated with tangential tractional force from the epiretinal membrane (ERM) with a disruption and separation of the retinal layer (typically at the Henle’s fiber layer level) is classified as ERM foveoschisis. Furthermore, macular pseudohole (MPH) is defined as a non-foveal involved ERM with a verticalized foveal edge [10, 11].

The differences in the hypothesized pathophysiology could interfere with the surgical responses. Thus, in this study, we aimed to report both functional and OCT anatomical outcomes (monitored by SD-OCT) of patients with ERM foveoschisis with visual impairment who underwent pars plana vitrectomy (PPV) with the internal limiting membrane (ILM) peeling and air tamponade.

## Materials and methods

### Study population

The study was conducted in accordance with the tenets of the Declaration of Helsinki, and the protocol was approved by the Ethics Committee, Faculty of Medicine, Chiang Mai University. Medical records of consecutive patients diagnosed as ERM and LMH with progressive visual impairment who underwent PPV between November 2017 and October 2018 were retrospectively reviewed. The ERM foveoschisis was diagnosed with OCT characteristics in accordance with the OCT-based definition proposed by Hubschman et al. Only the patients with the agreements by two opthalmologists (CP and SC) on the OCT findings were included in the study. The OCT criteria for ERM foveoschisis were as follows: 1) an irregularity of inner foveal contour with contractile ERM and 2) a presence of intraretinal schisis at the level of Henle’s fiber layer (a separation of outer plexiform layer (OPL) from outer nuclear layer (ONL)). The additional significant characteristics included the presence of intraretinal cyst within the inner nuclear layer (INL), retinal thickening, and retinal wrinkling [10].

Exclusion criteria were eyes with pre-operative OCT images classified as primary LMH (presence of irregular inner foveal contour, disruption of the inner retinal layer, and an undermined foveal cavitation edge), and other concurrent macular conditions leading to similar inner foveal contour abnormalities such as age-related macular degeneration, diabetic retinopathy and/or diabetic macular edema, retinal vascular occlusion, and uveitis. Additionally, eyes with other concurrent ophthalmic conditions precluding VA assessment such as advanced glaucoma, myopia more than − 6.0 diopters spherical equivalent, and previous PPV were also excluded. If both eyes were affected, the eye with greater visual impairment was included in the study. All participants provided written informed consents before the operation.

### Surgical techniques

A standard 23G, 3-port vitrectomy was performed by one retina specialist (JC). In addition, a posterior vitreous detachment was induced if indicated. A brilliant blue dye was used to stain for facilitating ERM and ILM peeling (at least two disc-diameters toward the major vascular arcade around the fovea). At the final step, fluid-air exchange was performed. All patients maintained a face-down position for 24 h.

### Spectral-domain optical coherence tomography retinal images

The cross-sectional eye-tracking macular images were obtained from Spectralis HRA SD-OCT (Spectralis, Heidelberg Engineering, Heidelberg, Germany) using a 20° × 20° scan area consisting of 49 raster B-scan lines with a high-resolution mode (1024 A-scans per B-scan line). In addition, a detailed 15° × 5° macular scan consisting of 49 raster B-scan lines was also performed. Nine averaged images of automatic real-time (ART) function were set to enhance an image resolution. All OCT images were analyzed with the Heidelberg eye explorer version 1.10.2.0 and the HRA/Spectralis viewing module version 6.9.5.0. The software calliper function was used to measure the minimum central foveal thickness (the vertical distance from the bottom of schisis to the top surface of the retinal pigment epithelium) and the schisis diameter (the horizontal distance of the schisis at the junction of OPL and ONL) before the operation. In addition, the presence of epiretinal proliferation, intraretinal cyst, and photoreceptor outer segment disruption was also determined. Post-operation, anatomical outcome and central foveal thickness were evaluated. A successful anatomical restoration was defined as the absence of inner foveal break with the disappearance of intraretinal schisis. The OCT grading was also performed by two ophthalmologists (CP and SC).

All patients were evaluated for demographic data, including age and gender. In addition, at baseline and each follow-up visit (1-, 3-, 6-, and 12-month post-operation), the clinical ophthalmologic characteristics (Snellen best-corrected VA (BCVA) and slit-lamp biomicroscopy with dilated fundus findings) and ophthalmologic images (color, infrared and red-free fundus photographs, and OCT image) were analyzed. Moreover, intra-operative and post-operative complications were noted.

### Statistical analysis

The continuous variables were presented as mean (standard deviation, SD), and the categorical variables were presented as frequency (percentage). The Snellen fraction VA was converted to the LogMAR (the logarithm of the minimum angle of resolution) unit for statistical analysis. To correct the repeated measurements of VA and central foveal thickness, multilevel analysis adjusted by baseline VA was performed. The correlation between VA and minimum central foveal thickness was estimated by Pearson correlation. All the data were analyzed with the STATA version 16.0 software. The statistical significance was considered as a *P*-value less than 0.05.

## Results

Overall, 14 patients (14 eyes), defined as ERM foveoschisis, were included in this study. The mean (SD) age was 67.9 (7.9) years (range 52 to 81 years). At baseline, the mean (SD) VA was 0.6 (0.1) LogMAR units (Snellen equivalent 20/80), range 0.5 to 0.8 LogMAR units (Snellen equivalent 20/63 to 20/125). None had epiretinal proliferation. Other baseline characteristics of the patients are summarized in Table 1.

**Table 1:** Baseline Clinical Characteristics of Patients with Epiretinal Membrane Foveoschisis Underwent Pars Plana Vitrectomy

Characteristics	
Age (year), mean (SD)	67.9 (7.9)
Female (N, %)	12 (85.7)
Laterality, (N, %)	8 (57.14
Phakia (N, %)	12 (85.7)
Axial length (millimeter), mean (SD)	22.8 (0.4)
Outer segment disruption, (N, %)	1 (7.1)
Intraretinal schisis diameter (micron), mean (SD)	1138.5 (564.3)

*Abbreviation*: *SD* standard deviation

For post-operation, all patients completed the follow-up visits at month 1-, 3-, 6-, and 12, respectively. Significant VA improvements were observed at all follow-up visits (*P*-values between < 0.001 and 0.011) compared to baseline. Of note, at 6-month post-operation, three patients developed progression of a cataract requiring surgical intervention. After the cataract extraction with intraocular lens implantation, the mean (SD) VA improved to 0.37 (0.21) LogMAR at the 12-month follow-up visit (Table 2). For the VA changes at 12 months post-operation, there were vision improvements of ≥3 lines in 8 (57.2%) patients, vision improvements of 1 or 2 lines in 2 (14.3%) patients, vision remained at the same line of pre-operation in 3 (21.4%) patients, and vision decreased by 1 line in 1 (7.1%) patient. Baseline intraretinal schisis distance was not associated with 12-month VA level (*P*-value = 0.09). In addition, a non-significant correlation between the minimum central foveal thickness and VA was observed at each follow-up visit (*P*-values from 0.21 to 0.86).

**Table 2:** Changes in Visual Acuity and Minimum Central Foveal Thickness by Follow-up Visits of The Patients with Epiretinal Membrane Foveoschisis Underwent Pars Plana Vitrectomy

	Visual Acuity (LogMAR)		Minimum Central Foveal Thickness (Micron)	
	Mean (SD)	***P***-value	Mean (SD)	***P***-value
**Baseline**	0.60 (0.13)	Reference	191.09 (48.74)	Reference
**Month 1**	0.49 (0.13)	0.011	283.79 (48.77)	< 0.001
**Month 3**	0.43 (0.14)	< 0.001	264.14 (48.99)	< 0.001
**Month 6**	0.45 (0.16)	0.003	244.93 (49.72)	< 0.001
**Month 12**	0.37 (0.21)	< 0.001	247.93 (52.55)	< 0.001

*Abbreviations*: *LogMAR* logarithm of the minimum angle of resolution, *SD* standard deviation

**Fig. 1:**
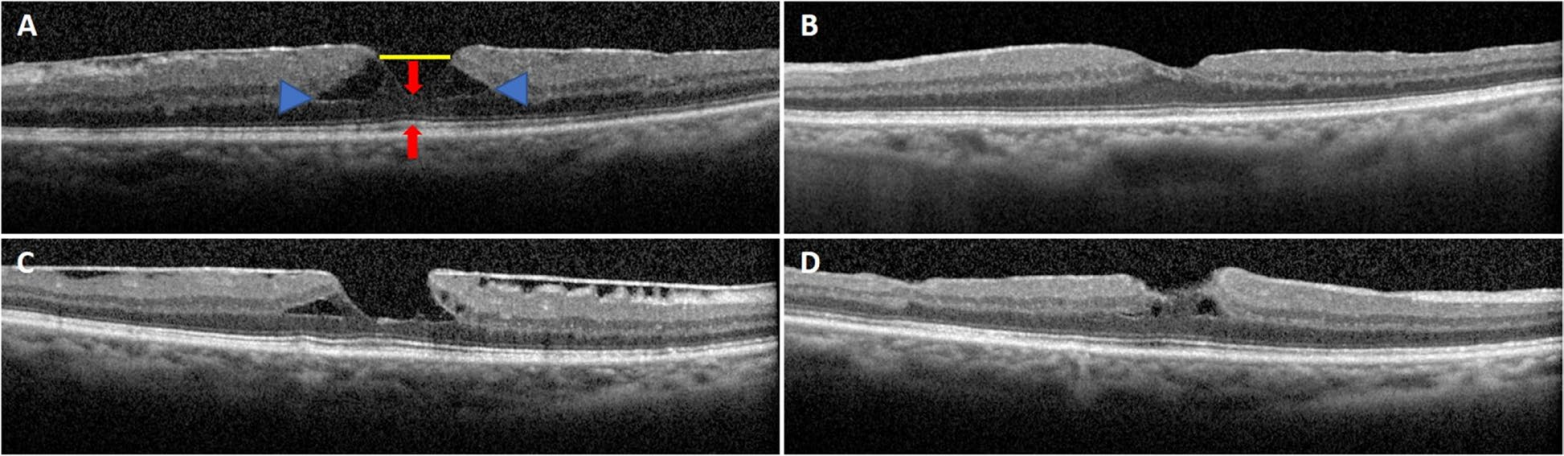
**A**, **B** Pre- and post-operative optical coherence tomography images of a 56-year-old woman with a pre-operation visual acuity of 20/80 who achieved an anatomical foveal restoration with visual improvement to 20/30 at the 3-month follow-up visit. Blue arrowheads indicate intra-retinal schisis diameter, red arrows indicate minimum central foveal thickness, and the horizontal yellow line indicates inner foveal break. **C**, **D** Pre- and post-operative optical coherence tomography images of a 61-year-old woman with pre-operative visual acuity of 20/80 who had persistent intraretinal schisis with stabilized vision (20/80) at 12-month follow-up visit

**Fig. 2:**
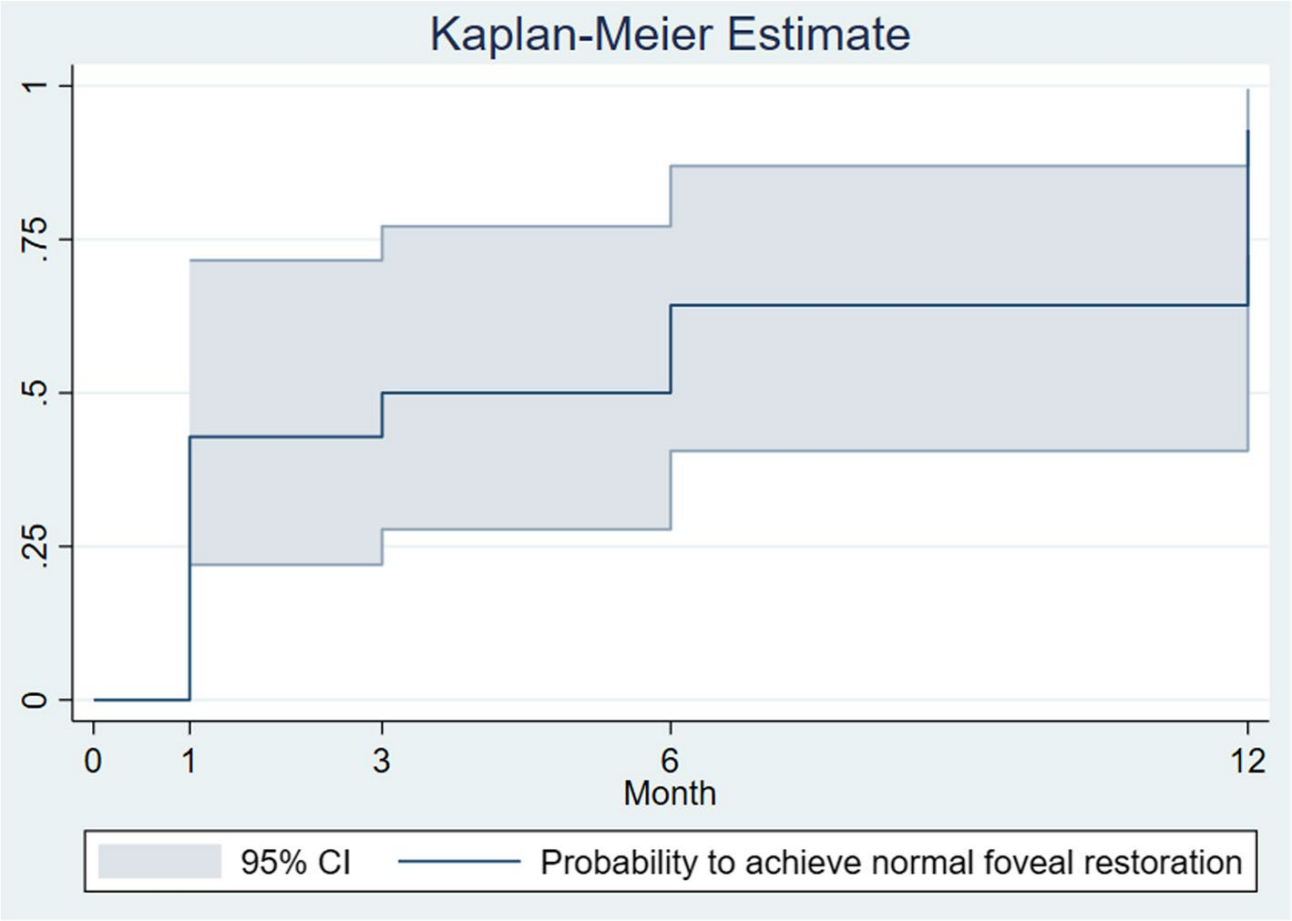
Kaplan-Meier estimate of the probability to achieve anatomical foveal restoration of Epiretinal Membrane Foveoschisis Patients Underwent Pars Plana Vitrectomy

Regarding the anatomical outcomes, a significant increase in minimum central foveal thickness compared to baseline was observed at all follow-up visits (all *P*-values < 0.001, Table 2). Thirteen of 14 (92.9%) patients had a successful anatomical foveal restoration, while 1 patient had an improved but persistent intraretinal schisis at the end of the 12-month follow-up (Fig. 1). The median time to attain an anatomical foveal restoration was 3 months. The Kaplan-Meier estimate for the probability of foveal restoration is illustrated in Fig. 2. As noted, the probability of foveal restoration at month 12 was 96% (95% CI: 77.2 to 99.5%). The persistence of photoreceptor outer segment disruption was observed in 1 patient.

### Surgical complications

No intra-operative complication was noted. No patient developed a full-thickness macular hole or retinal detachment.

## Discussion

The clinical course and surgical outcome of patients with irregular foveal contour and separation of the inner retinal layer have been reported. However, with variation in diagnostic definition, nomenclature (LMH, MPH, etc.), degree of high myopia, and heterogeneity in the OCT characteristics, some significant clinical results remain inconclusive due to incomparable data between studies [4, 6, 7, 9, 12–18]. Consequently, Hubschman et al. have recently proposed a consensus OCT-based definition that clearly differentiates ERM-associated foveoschisis from MPH and LMH [10]. Even though the definite pathogenesis remains unclear, a potential mechanism causing intraretinal splitting between OPL and ONL in ERM foveoschisis is a contraction of the eccentric perifoveal membrane. Therefore, surgical prognosis after vitrectomy to remove the contractile component is likely to have a different response pattern from those with LMH (which is presumably associated with a degenerative mechanism).

In previous literature, a spontaneous detachment of ERM with a restoration of foveal contour was reported but with an infrequent incidence [19, 20]. In conjunction with a relative stabilized natural course, a surgical management is mainly indicated for those patients who experience progressive visual and/or anatomical deterioration. Several authors investigated beneficial effects of vitrectomy for conditions presenting with ERM and intraretinal schisis-like separation (defined as tractional LMH or MPH with stretched/cleavage edge) in terms of post-operative BCVA and OCT foveal structures [15, 21–23]. Among those, Gaudric et al. reported an overall visual improvement, assessed at 3 months or more post-operation, in both MPH with straight and cleavage foveal edge that had initial VA worse than 20/40 [15]. Coassin et al. also observed a significant visual improvement following vitrectomy, assessed at 6 months and at the final follow-up visit, for symptomatic LMH with a tractional schisis-like type (but not for degeneration) [9].

Recently, a study by Omoto et al., with an application of a consensus OCT definition, has demonstrated a significant visual gain at 3 months and at the final visit for ERM foveoschisis patients who underwent vitrectomy. For the LMH group, a significant visual improvement was observed only at the final visit [21].

Consistent to others, a significant post-operative visual improvement could be rapidly achieved at 1 month and maintained until the end of the study period at 12 months in this study, compared to baseline. None experienced a decrease in vision of two or more lines or developed a new photoreceptor outer segment disruption after the operation. Visual improvement of 3 lines or more was present in 57% of patients. These overall favorable visual outcomes after vitrectomy in ERM foveoschisis may partly be attributed to the preserved photoreceptor outer segment in this condition. Thus, the PPV with ERM and ILM peeling showed a beneficial role for visual improvement in symptomatic ERM foveoschisis patients.

Regarding anatomical structures on OCT, a high success rate for foveal restoration following vitrectomy was previously described in publications [8, 15, 23]. Gaudric et al. reported a disappearance/attenuation of stretched foveal edge in 17 of 19 eyes when assessed at the 3-month post-operation visit [15]. Similarly, Figueroa et al. reported a successful anatomical restoration in 72 of 77 tractional LMH cases with a mean time to restoration of 3.3 months (over a mean observation of 30.8 months) [23]. In this study, a median time to foveal restoration was 3 months and more than 90% of patients achieved normalized foveal contour at the 12-month follow-up. Nonetheless, even with a remarkable anatomical improvement, the association between anatomical restoration and post-operative VA was reported to be non-significant in several studies [23]. Likewise, this study found that baseline macular morphology and normalized characteristics did not significantly influence visual outcome. However, larger prospective investigations are required to explore the definite relationship between anatomical restoration and functional improvement following vitrectomy in ERM foveoschisis.

Even though a standard recommendation for timing of surgical management and the surgical technique for ERM foveoschisis remains inconclusive, this study reports promising visual and anatomical restoration following vitrectomy with ERM and ILM peeling over a 1-year study period in symptomatic patients. In addition, favorable functional and anatomical responses following conventional PPV with ERM and ILM peeling for ERM foveoschisis (tractional LMH) were demonstrated (in accordance with most publications), in contrast to the LMH cases where surgical efficacy was variously reported depending on surgical techniques [19, 23–27]. Also, the benefits of performing intravitreal tamponade at the end of surgery are still under-explored.

The limitations of the study included a retrospective cohort design, a small sample size, a short follow-up period, and a lack of comparison with other similar morphological-looking conditions. However, its strength is the uniform OCT characteristics of the patients with exact follow-up timepoints which revealed the trends of visual and anatomical improvement within the study period. The information is useful for counselling and monitoring patients.

In conclusion, this study provides an evidence that vitrectomy with ERM and ILM peeling is an effective treatment to restore both functional and anatomical outcomes in patients with ERM foveoschisis with visual deterioration. However, further studies with larger sample sizes and longer follow-up periods are required.

### Summary

Par plana vitrectomy with ERM and ILM peeling is an efficient procedure for ERM foveoschisis with visual impairment and/or anatomical progression. Nearly two thirds of the patients achieved promising visual outcomes with normalized macular structures on OCT.

## Data Availability

The datasets generated and/or analyzed during the current study are not publicly available due to limitations of ethical approval involving the patient data and anonymity but are available from the corresponding author on reasonable request.
